# Supplementary material for: Transcriptomics of type 2 diabetic and healthy human neutrophils
Source: BMC Immunol. 2021 Jun 16;22:37. doi: 10.1186/s12865-021-00428-6 (PMC8207744; doi:10.1186/s12865-021-00428-6)
Supplement: Supplementary file 1 — Additional file 1: Supplementary Table S1. Over-represented KEGG pathways. Supplementary Table S2. Top 5 statistically significant over-represented diseases. Supplementary Table S3. Average cytokine levels (pg/mL) by matched RvE1 treatment of T2D (N = 3) and healthy (N = 3) neutrophils in cell culture. Supplementary Table S4. Average cytokine levels (pg/mL) by differing RvE1 treatment of T2D (N = 3) and healthy (N = 3) neutrophils in cell culture. Supplementary Fig. S1. Principal component analysis (PCA) plot of type 2 diabetic and healthy subjects. Supplementary Fig. S2. Neutrophil gene expression in type 2 diabetic versus healthy subjects for all 50 significant genes. Supplementary Fig. S3. Top 50 significant neutrophil genes differentially expressed in type 2 diabetic and healthy subjects by top biological pathways. Supplementary Fig. S4. Neutrophil gene expression of type 2 diabetic and healthy subjects by biologically relevant KEGG pathways. [file 12865_2021_428_MOESM1_ESM.docx]

**Supplementary Material**

**Title:** Transcriptomics of Type 2 Diabetic and Healthy Human Neutrophils

**Authors:** Sarah E. Kleinstein, Jamison McCorrison, Alaa Ahmed, Hatice Hasturk, Thomas E. Van Dyke, Marcelo Freire.

**Supplementary Tables: 4**

**Supplementary Figures: 4**

**Supplementary Tables:**

**Supplementary Table S1.** Over-represented KEGG pathways.

| **Pathway Name** | **p-value** | **p-value (FDR)** | **p-value (Bonferroni)** |
| --- | --- | --- | --- |
| Sphingolipid metabolism | 2.52E-04 | 0.02 | 0.02 |
| Ether lipid metabolism | 0.004 | 0.14 | 0.28 |
| Phospholipase D signaling pathway | 0.02 | 0.24 | 1 |
| Fc gamma R-mediated phagocytosis | 0.02 | 0.24 | 1 |
| Glycerophospholipid metabolism | 0.02 | 0.24 | 1 |
| Colorectal cancer | 0.03 | 0.24 | 1 |
| Sphingolipid signaling pathway | 0.03 | 0.24 | 1 |
| Viral carcinogenesis | 0.03 | 0.24 | 1 |
| Tuberculosis | 0.03 | 0.24 | 1 |
| Transcriptional misregulation in cancer | 0.03 | 0.24 | 1 |
| Fluid shear stress and atherosclerosis | 0.04 | 0.24 | 1 |
| Complement and coagulation cascades | 0.05 | 0.24 | 1 |
| MicroRNAs in cancer | 0.05 | 0.24 | 1 |

Uncorrected p < 0.05 (before rounding) considered over-represented; Bonferroni or FDR-corrected p-value < 0.05 considered statistically significant. FDR, false discovery rate.

**Supplementary Table S2.** Top 5 statistically significant over-represented diseases.

| **Disease** | **No. Genes (DE/All)** | **p-value** | **FDR-adjusted p-value** |
| --- | --- | --- | --- |
| Citrullinemia | 1 / 1 | 0.003 | 0.03 |
| Paget's disease of bone and related disorders | 1 / 2 | 0.006 | 0.03 |
| Endometriosis | 1 / 3 | 0.009 | 0.03 |
| Epidermolysis bullosa, junctional | 1 / 4 | 0.01 | 0.03 |
| Avascular necrosis of the femoral head /  Osteonecrosis of the femoral head | 1 / 4 | 0.01 | 0.03 |

Uncorrected p < 0.05 considered over-represented; FDR-corrected p-value < 0.05 considered statistically significant. DE, differentially expressed; FDR, false discovery rate.

**Supplementary Table S3.** Average cytokine levels (pg/mL) by matched RvE1 treatment of T2D (N = 3) and healthy (N = 3) neutrophils in cell culture.

| **Cytokine** | **Mean Healthy (0 nM RvE1)** | **Mean T2D (0 nM RvE1)** | **p-value** | **Mean Healthy (1 nM RvE1)** | **Mean T2D (1 nM RvE1)** | **p-value** | **Mean Healthy (10 nM RvE1)** | **Mean T2D (10 nM RvE1)** | **p-value** | **Mean Healthy (100 nM RvE1)** | **Mean T2D (100 nM RvE1)** | **p-value** |
| --- | --- | --- | --- | --- | --- | --- | --- | --- | --- | --- | --- | --- |
| **MIP-1α** | 10 | 11.2 | 0.67 | 11.2 | 15.7 | 0.19 | 18.0 | 21.5 | 0.80 | 34.2 | 22.5 | 0.71 |
| **IL-4** | 155 | 155 | - | 155 | 157.2 | 0.37 | 155 | 155 | - | 155 | 155 | - |
| **IL-8** | 394.2 | 310.4 | 0.68 | 416.4 | 322.7 | 0.61 | 385.9 | 334.2 | 0.79 | 554.8 | 338.7 | 0.23 |
| **MIP-1β** | 80.4 | 74 | 0.79 | 83.5 | 82.2 | 0.96 | 115.5 | 93.7 | 0.56 | 239.9 | 137.4 | 0.52 |
| **P-Selectin** | 1219 | 1737 | 0.47 | 1219 | 1937 | 0.35 | 1090 | 1578 | 0.37 | 1511 | 1751 | 0.76 |
| **sICAM-1** | 555.8 | 493.1 | 0.61 | 469.3 | 481.2 | 0.79 | 469.3 | 445.6 | 0.50 | 516.9 | 445.6 | - |
| **TNF-α** | 15 | 20.3 | 0.52 | 15 | 17.6 | 0.64 | 22.9 | 21.7 | 0.74 | 24.6 | 19.3 | 0.68 |
| **IL-1α** | 6.3 | 6.2 | 0.86 | 6.7 | 6.2 | 0.73 | 6.1 | 6.2 | 0.95 | 10.8 | 6.2 | 0.15 |

*p*-values calculated by unpaired t-tests, where possible. Significance threshold: p < 0.05. RvE1, resolvin E1; T2D, type 2 diabetes.

**Supplementary Table S4.** Average cytokine levels (pg/mL) by differing RvE1 treatment of T2D (N = 3) and healthy (N = 3) neutrophils in cell culture.

| **Cytokine** | **Mean Healthy (0 nM RvE1)** | **Mean Healthy (100 nM RvE1)** | **p-value** | **Mean T2D (0 nM RvE1)** | **Mean T2D (100 nM RvE1)** | **p-value** | **Mean Healthy (0 nM RvE1)** | **Mean T2D (100 nM RvE1)** | **p-value** |
| --- | --- | --- | --- | --- | --- | --- | --- | --- | --- |
| **MIP-1α** | 10 | 34.2 | 0.39 | 11.2 | 22.5 | 0.46 | 10 | 22.5 | 0.42 |
| **IL-4** | 155 | 155 | - | 155 | 155 | - | 155 | 155 | - |
| **IL-8** | 394.2 | 554.8 | 0.47 | 310.4 | 338.7 | 0.84 | 394.2 | 338.7 | 0.79 |
| **MIP-1β** | 80.4 | 239.9 | 0.29 | 74 | 137.4 | 0.38 | 80.4 | 137.4 | 0.43 |
| **P-Selectin** | 1219 | 1511 | 0.38 | 1737 | 1751 | 0.99 | 1219 | 1751 | 0.47 |
| **sICAM-1** | 555.8 | 516.9 | 0.88 | 493.1 | 445.6 | 0.42 | 555.8 | 445.6 | 0.67 |
| **TNF-α** | 15 | 24.6 | 0.33 | 20.3 | 19.3 | 0.93 | 15 | 19.3 | 0.71 |
| **IL-1α** | 6.3 | 10.8 | 0.14 | 6.2 | 6.2 | 0.95 | 6.3 | 6.2 | 0.94 |

*p*-values calculated by unpaired t-tests, where possible. Significance threshold: p < 0.05. RvE1, resolvin E1; T2D, type 2 diabetes.

**Supplementary Figures:**

**
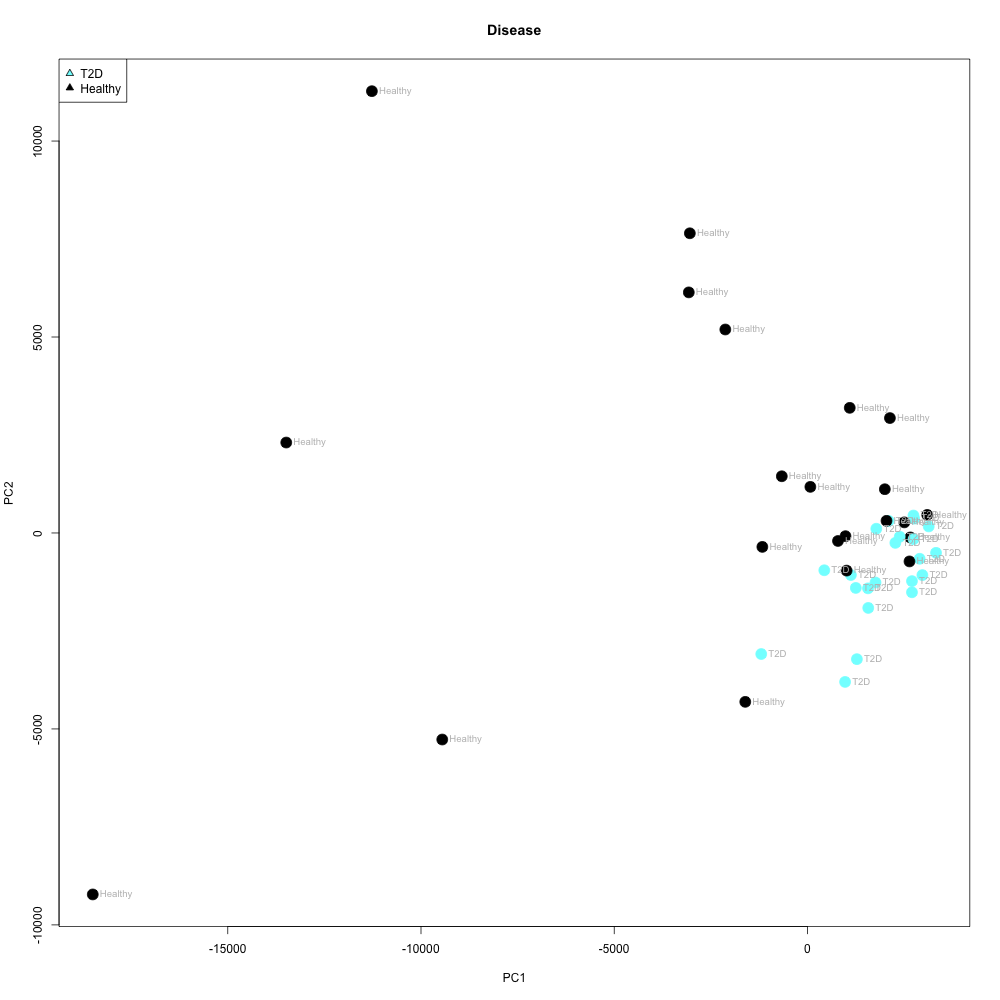
**

**Supplementary Figure S1. Principal component analysis (PCA) plot of type 2 diabetic and healthy subjects**. EIGENSTRAT PCA plot of the two main axes of variation (PC1, x-axis; PC2, y-axis) for gene expression results, color-coded by disease: blue = type 2 diabetic; black = healthy. T2D, type 2 diabetes; PC1, principal component 1; PC2, principal component 2.

**
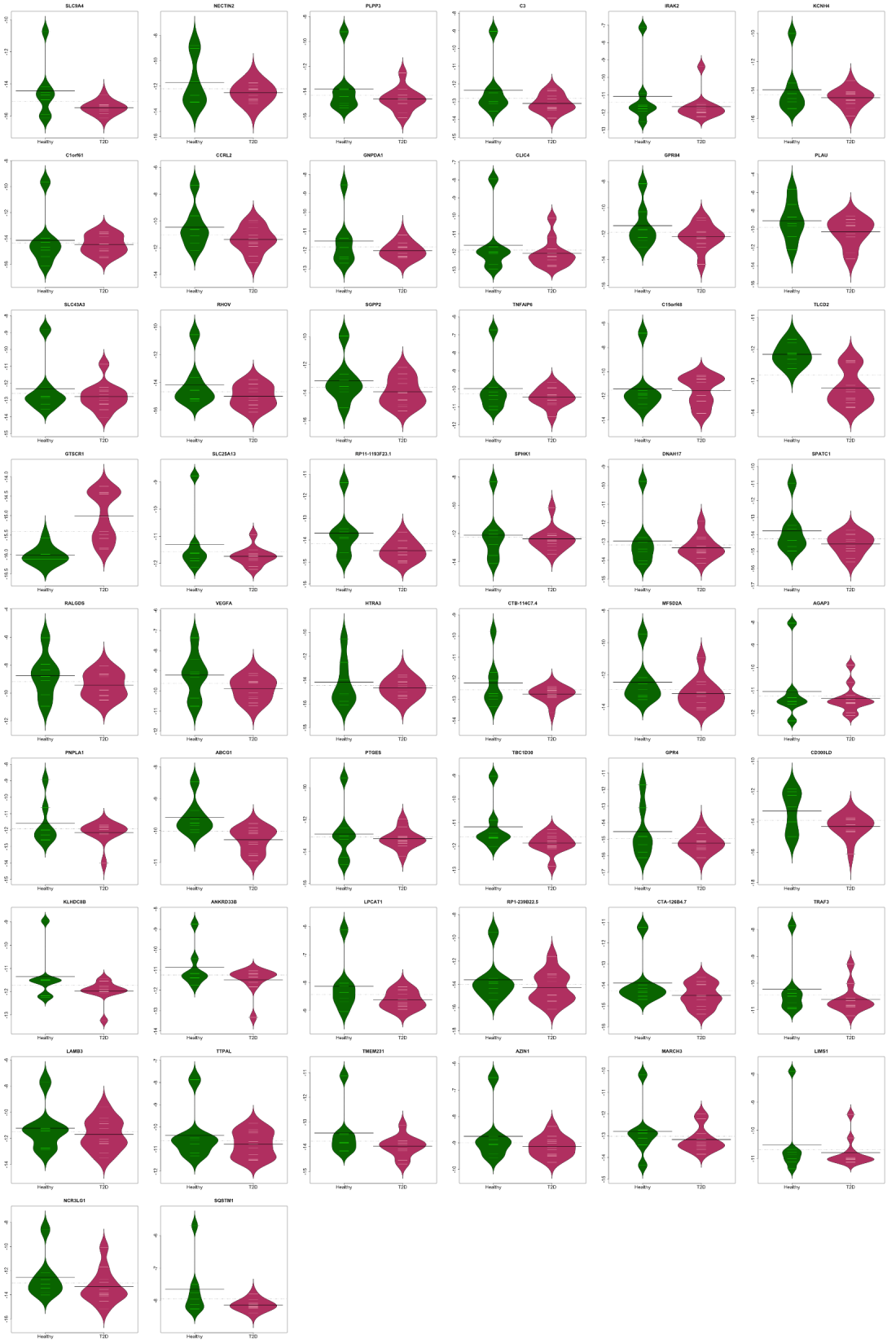
**

**Supplementary Figure S2. Neutrophil gene expression in type 2 diabetic versus healthy subjects for all 50 significant genes.** Bean plots of the ln-transformed relative abundance of neutrophil gene expression (line at mean) of all 50 significant (FDR-corrected p < 0.05) differentially expressed genes between T2D and healthy subjects. T2D, type 2 diabetes.


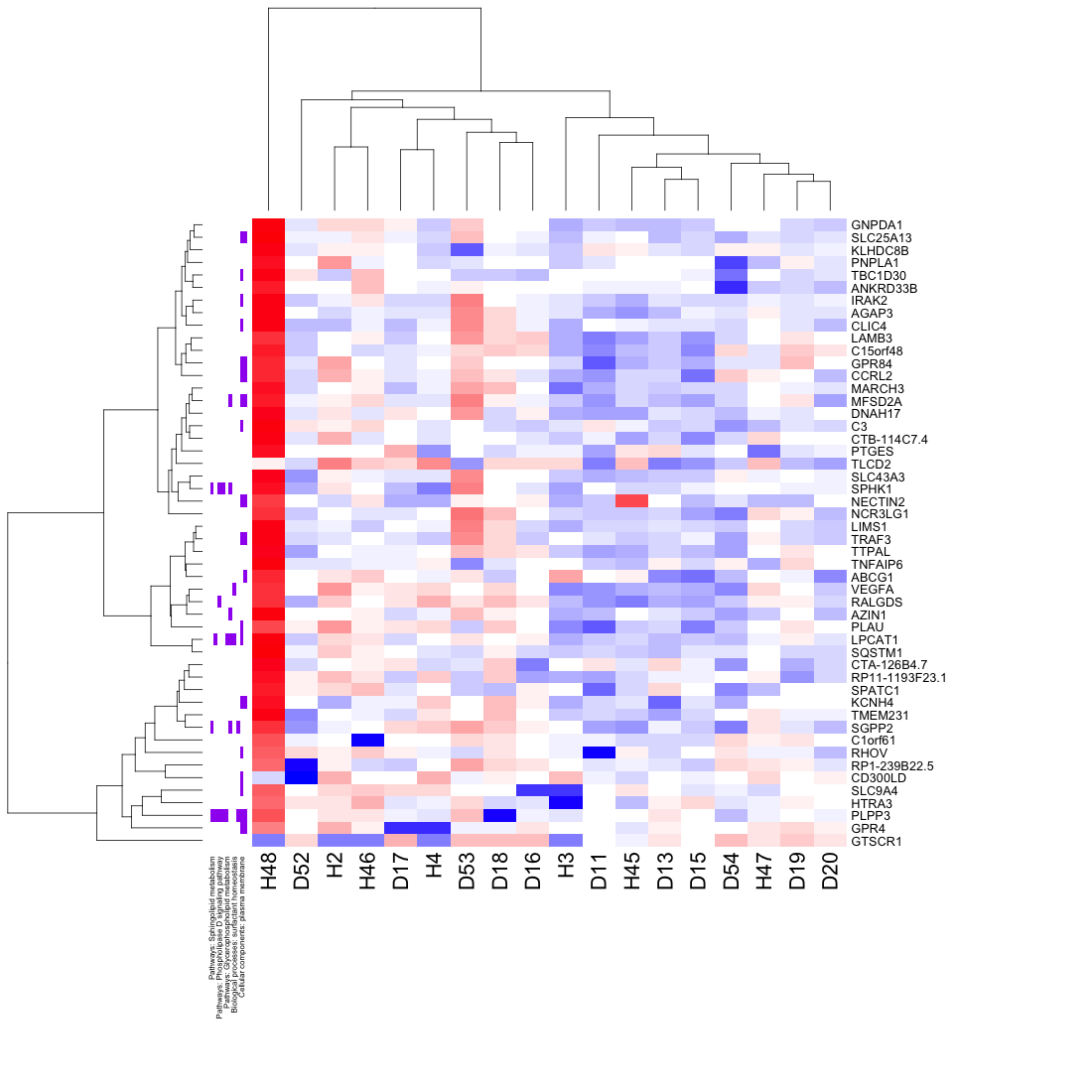

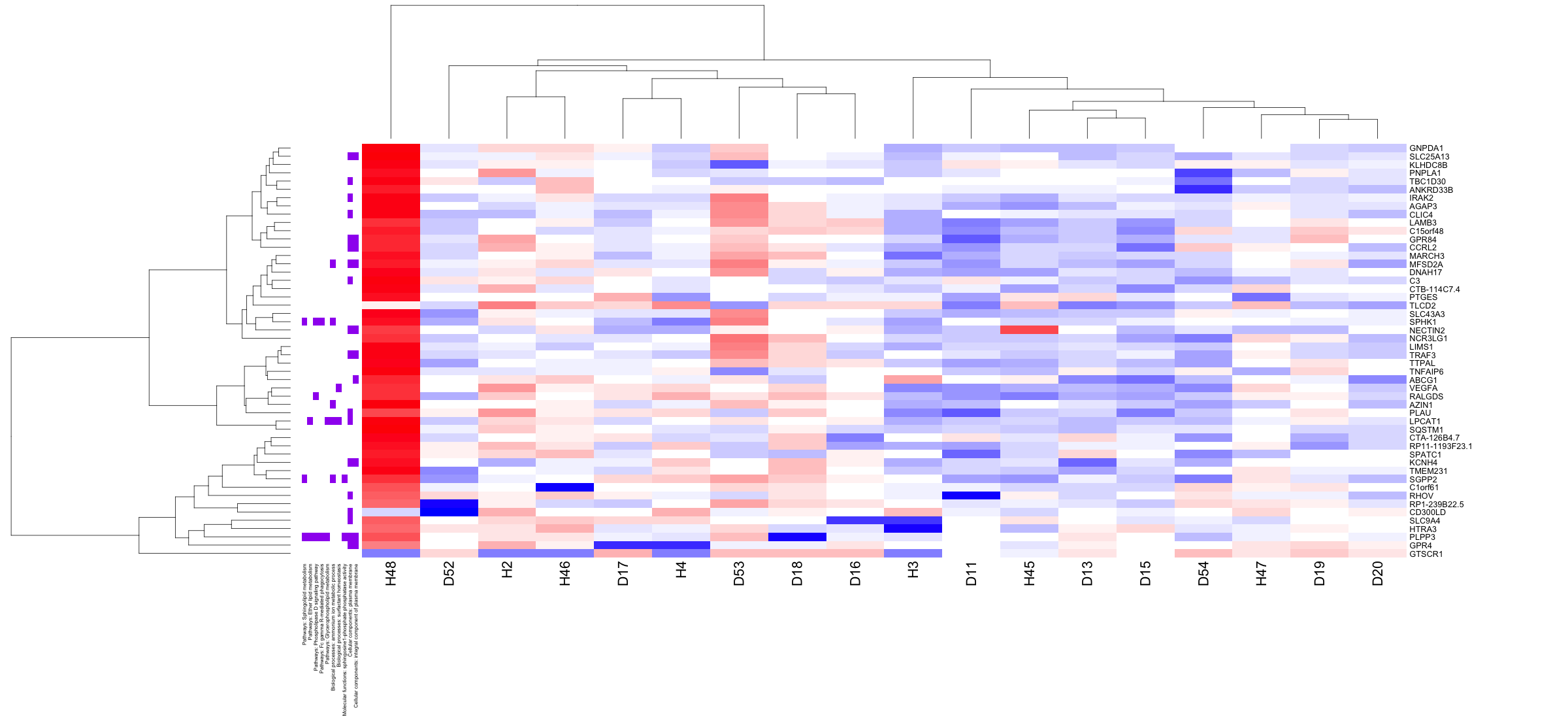

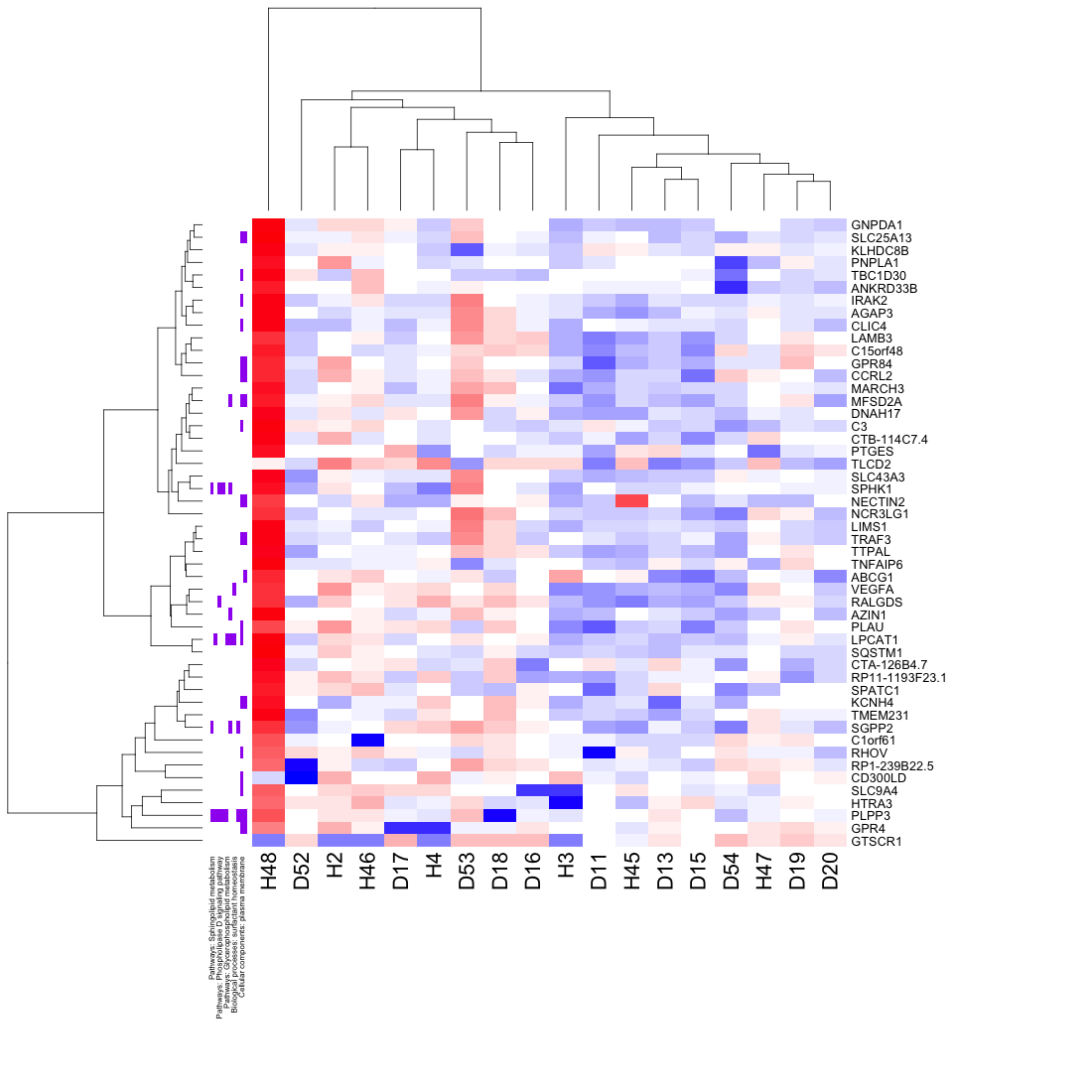


**Supplementary Figure S3. Top 50 significant neutrophil genes differentially expressed in type 2 diabetic and healthy subjects by top biological pathways.** RNA-seq transcript expression of T2D (D; N = 11) vs. healthy (H; N =7) subject neutrophils. Heatmap of T2D and healthy gene expression by over-represented KEGG and Gene Ontology (GO) pathways (L to R: sphingolipid metabolism, ether lipid metabolism, phospholipase D signaling pathway, Fc gamma R-mediated phagocytosis, glycerophospholipid metabolism, ammonium ion metabolic process, surfactant homeostasis, sphingosine-1-phosphate-phosphatase activity, plasma membrane, integral component of plasma membrane) across all 50 statistically significant differentially expressed genes (FDR-corrected p < 0.05). Blue indicates decreased and red indicates increased gene expression.

**
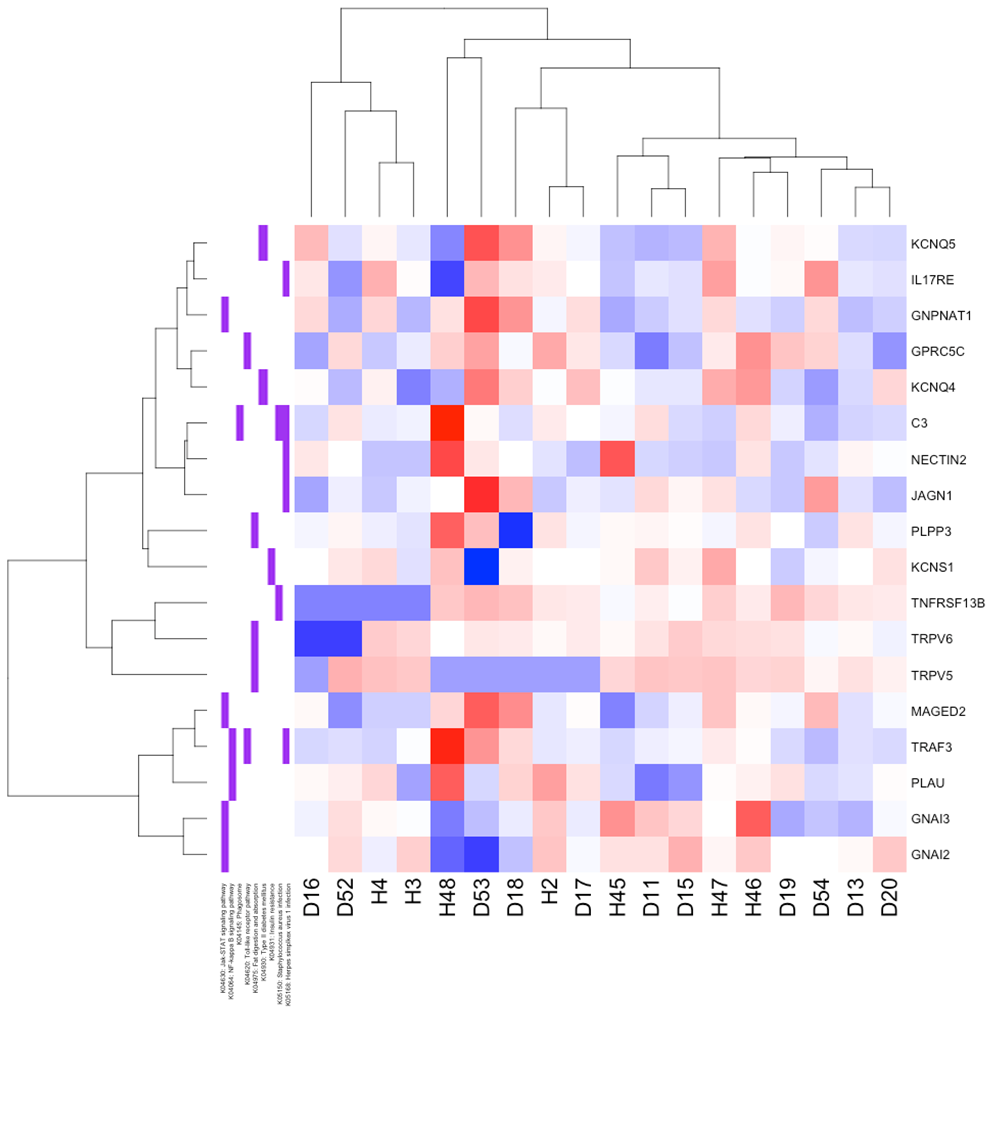
**

**Supplementary Figure S4. Neutrophil gene expression of type 2 diabetic and healthy subjects by biologically relevant KEGG pathways.** RNA-seq transcript expression of T2D (D; N = 11) vs. healthy (H; N = 7) subject neutrophils. Heatmap of T2D and healthy gene expression by biologically relevant KEGG pathways (L to R: Jak-STAT signaling pathway, NFκB signaling pathway, Phagosome, Toll-like receptor pathway, Fat digestion and absorption, Type II diabetes mellitus, Insulin resistance, *Staphylococcus aureus* infection, Herpes simplex virus 1 infection) and all differentially expressed genes in those pathways. Blue indicates decreased and red indicates increased gene expression.
